# A New Species of *Ellipsomyxa* (Myxozoa: Ceratomyxidae) from the Gall Bladder of the Amazonian Catfish *Ageneiosus inermis* (Siluriformes: Auchenipteridae)

**DOI:** 10.1007/s11686-026-01290-5

**Published:** 2026-05-11

**Authors:** Caio Vitor da Conceição Costa, Flávia Letícia Gurjão de Andrade, Mikaela Vivian Pereira Andrade, João Victor Morais Valente, Debora Sayumi Doami Melo, André Luiz Alves de Sá, Sávio Lucas de Matos Guerreiro, Diehgo Tuloza da Silva, Edilson Rodrigues Matos, Igor Guerreiro Hamoy

**Affiliations:** Socio-Environmental and Hydrological Resources Institute, Laboratory for Applied Genetics, Federal Rural University of Amazonia, Belém, Pará Brazil

**Keywords:** Myxosporea, Phylogeny, Parasite diversity, Cnidarians, Fish parasites

## Abstract

The fish fauna of the basin of the Amazon River is one of most diverse of any region on Earth and hosts a parasite fauna that is poorly studied. One group of fish parasites is the Myxosporea, a class of the phylum Cnidaria, which are obligate endoparasites. The present study investigated myxozoans found in specimens of the Neotropical catfish *Ageneiosus inermis* collected in Marajó Bay, off Humaitá Beach. A total of 25 *A. inermis* specimens were captured and anaesthetised for necropsy, including the gallbladder, liver and muscle tissue, which were prepared for light microscopy, molecular biology and histology. Plasmodia and spores with morphology typical of the genus *Ellipsomyxa* were observed in the gallbladders of 14 of the individuals. The disporic plasmodia contain two mature spores measuring 6.6 μm × 4.0 μm, on average, with subterminal polar capsules. The analysis of a partial sequence of the 18 S rDNA gene, together with the morphological and morphometric characteristics of the specimens, supported conclusively the description of a new species of the genus *Ellipsomyxa*. This is the first record of a species of this genus parasitising *A. inermis*. This represents the first record of a species of this genus parasitizing *A. inermis* and describes a new taxon.

## Introduction

The Amazon basin stretches across nine countries – Brazil, Peru, Colombia, Bolivia, Ecuador, Venezuela, Guiana, Suriname, and French Guiana – and is recognised as the world’s largest hydrographic basin, with a vast network of rivers and tributaries that contain an enormous diversity of fish species of inestimable ecological and economic relevance [1, 2]. *Ageneiosus inermis* (Linnaeus, 1766) is a species of catfish (Siluriformes), which is known in the Brazilian Amazon region as the mandubé, and is a member of the family Auchenipteridae. This species is amply distributed in the hydrographic systems of South America [3, 4]. It presents a high level of ecological adaptability and not only plays a prominent role in local trophic webs, but is also an important economic and social resource, especially for the riverside communities of the Amazon region, where it is exploited intensively by both artisanal and commercial fisheries [5].

The myxozoans are a highly diversified group of metazoan parasites, which have evolved endoparasitic interactions primarily with fish, although some species also infect other types of aquatic organism [6]. The diversity of myxozoans in fish, in both marine and freshwater environments, is still poorly understood, principally in the Amazon region, due primarily to the enormous diversity of fish found in this region [7, 8].

*Ellipsomyxa* [9] is one of the best-studied myxozoan genera, with approximately 24 species described to date, of which, seven were encountered in the Amazon region [10, 11]. The present study describes a new myxozoan species of the genus *Ellipsomyxa*, which was found in the gallbladder of specimens of *A. inermis* collected from the Amazon Coast. The new species was described based on the results of morphological, morphometric and phylogenetic analyses.

## Materials and Methods

### Host Collection

**Fig. 1:**
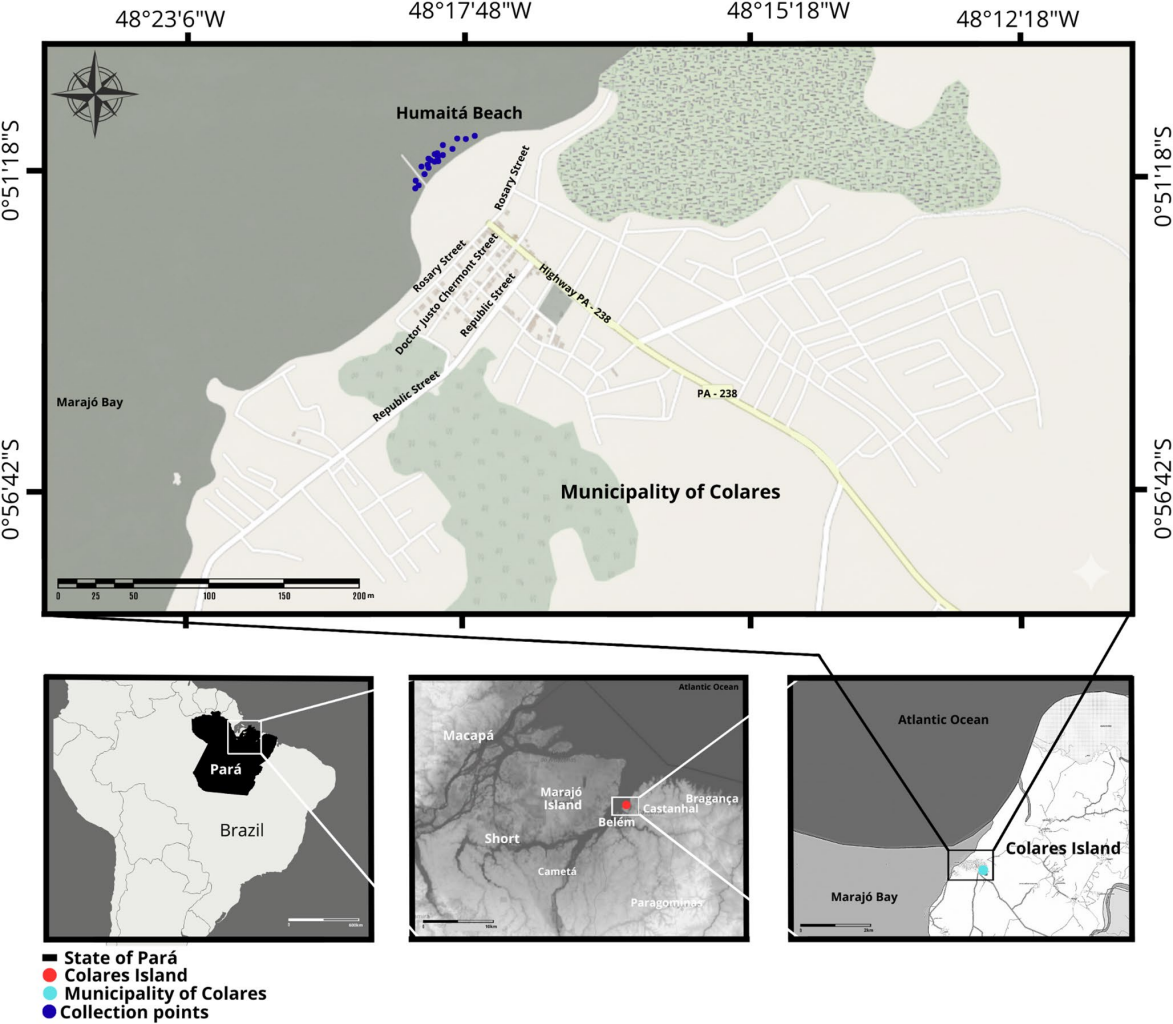
Location of georeferenced collection points in Marajó Bay, off Humaitá Beach, in the municipality of Colares, Pará State, Brazil

A total of 25 *A. inermis* specimens were collected in March 2022 in Marajó Bay, off Humaitá Beach, in the municipality of Colares, Pará State, Brazil (Fig. 1).

The specimens were transported alive to the former Carlos Azevedo Research Laboratory on the Belém campus of the Federal Rural University of Amazonia in coolers containing water from the original fish habitat, with artificial aeration.

The collection of the specimens was authorised by a special licence emitted by the Brazilian Institute for the Environment and Renewable Natural Resources (SISBIO ICMBio, licence 27119-1). The study was also conducted in accordance with protocol number 8,323,110,522/2022, which was approved by the Committee for the Ethical Use of Animals of the Federal Rural University of Amazonia.

### Morphological Analysis and Parasite Collection

Prior to necropsy, the fish in the aquariums were sacrificed with tricaine methanesulfonate (MS-222, SIGMA) at a concentration of 50 mg/L. Once they no longer exhibited gill movements and did not respond to stimuli, they were immediately subjected to myelotomy—a procedure consisting of the sectioning of the spinal cord to ensure the definitive interruption of nervous activity—and were subsequently necropsied.

A stereomicroscope was used during the necropsy to determine the presence of cysts indicative of microparasite infection on the body surface and in the internal organs. Small tissue fragments were collected from the hosts gallbladder and bile to assess the presence of spores. These fragments were sandwiched between a glass slide and coverslip containing a drop of water and then initially examined under a light microscope, using differential interference contrast (DIC) and photographed in fresh state.

After confirmation of the presence of spores, infected gallbladder and/or bile were subsequently fixed in Davidson’s solution (acetic acid, 95% ethanol, formaldehyde, and distilled water) at a ratio of nine parts solution to one part biological material (9:1) for a period of 24 h. The material was then dehydrated in a series of increasingly concentrated alcohols (70%, 80%, 90%, 100% I, 100% II, and 100% III), prior to clarification with xylene.

The samples were then embedded in blocks of paraffin using a Tissue Embedding Centre Micron EC350, for the extraction of 5 μm-thick histological sections using a traditional Carl Zeiss Hyrax M25 rotary microtome. These sections were transferred to glass slides, placed in a water bath and heated to 60 °C in a stove for 24 h for fixation. Following fixation, the slides were submitted to a series of staining processes using the Ziehl-Neelsen (ZN) technique. The slides were then photographed with a Zeiss Axiocam 512 color camera, attached to a Zeiss Primo Star microscope. Morphometric analysis was based on the measurements of 30 fresh spores, including the length of spore (LS) width of spore (WS), length of polar capsule (LP) and width of polar capsule (WP), reported as means, minimum, maximum, and standard deviations.

### Molecular and Phylogenetic Analyses

For the molecular analysis, fragments of parasitised tissue with microparasite spores were collected and fixed in 80% ethanol. The DNA was extracted using the PureLink^®^ Genomic DNA mini kit (Invitrogen, USA), following the manufacturer’s protocol for the extraction of “mammalian tissue and mouse/rat tail lysate”. This protocol was modified by extending the incubation with proteinase K throughout the night. Once extracted, the concentration of the DNA was measured using a BioDrop Duo spectrophotometer.

A fragment of the 18 S rDNA gene was amplified by polymerase chain reaction (PCR) using a two-step nested PCR approach on a LOCCUS TC 600 thermocycler. The first amplification step was performed with the primers 18E (CTGGTTGATCCTGCCAGT) and 18R (CTACGGAAACCTTGTTACG) [12], and the second amplification step employed the primers 18E–MC3 (GATTAGCCTGACAGATCACTCCACGA) and 18R–MC5 (CCTGAGAAACGGCTACCACATCCA) [13, 12]. PCR reactions were performed in a final volume of 25 µL, containing 0.5 µL of each primer, 10.5 µL of ultrapure water, 12.5 µL of 2× Taq DNA Polymerase Master Mix (1.5 mM MgCl₂), and 1 µL of genomic DNA (100 ng). The cycling conditions consisted of an initial denaturation at 95 °C for 5 min, followed by 35 cycles at 95 °C for 1 min, 56 °C for 1 min, and 72 °C for 1 min, with a final extension at 72 °C for 10 min. Aliquots of 5 µL of the PCR products were subjected to electrophoresis on 1% agarose gels prepared with 1× Tris–Borate–EDTA (TBE) buffer and stained with the nucleic acid dye DSView (SINAPSE). Successfully amplified products were purified according to the manufacturer’s protocol and sequenced using a Sanger Genetic Analyzer with the BigDye v3.1 Cycle Sequencing Ready Reaction Kit (Applied Biosystems).

After sequencing, the obtained DNA data were analyzed and edited in BioEdit 7.2 [14], including end trimming, removal of low-quality regions, and correction of ambiguous bases. Multiple sequence alignment was performed using ClustalW [15] with default parameters, followed by consensus sequence construction. The resulting consensus sequence was compared with sequences available in the NCBI (National Center for Biotechnology Information) databases using BLAST (Basic Local Alignment Search Tool) [16] against the nr/nt database to retrieve homologous sequences. For preliminary taxonomic identification, only alignments showing 100% query coverage were considered, ensuring analysis across the full length of the sequence.

For phylogenetic reconstruction, a dataset including other species of *Ellipsomyxa* as well as more distantly related species described in the literature was assembled to infer phylogenetic relationships. Sequences were aligned using ClustalW [15] implemented in Geneious R11 (v11.1.5) (https://www.geneious.com). Phylogenetic analysis was conducted using the Neighbor-Joining (NJ) method under the Tamura–Nei (TN93) evolutionary model, with branch support assessed by 1,000 bootstrap replicates. Tree rooting and graphical styling were finalized using iTOL (Interactive Tree of Life) v1.6.12 [17], with the genus *Myxidium* designated as the outgroup.

Genetic distance estimates for the partial 18 S rDNA fragment were obtained using PAUP* version 4.0b1 [18] applying the default p-distance parameter.

## Results

### Morphological Description of the Spores

Overall, 14 (56%) of the 25 *A. inermis* specimens examined in this study harbored spores and plasmodia of the genus *Ellipsomyxa*, both exhibiting variable sizes, distributed in both the bile and the gallbladder epithelium of the hosts.

**Fig. 2:**
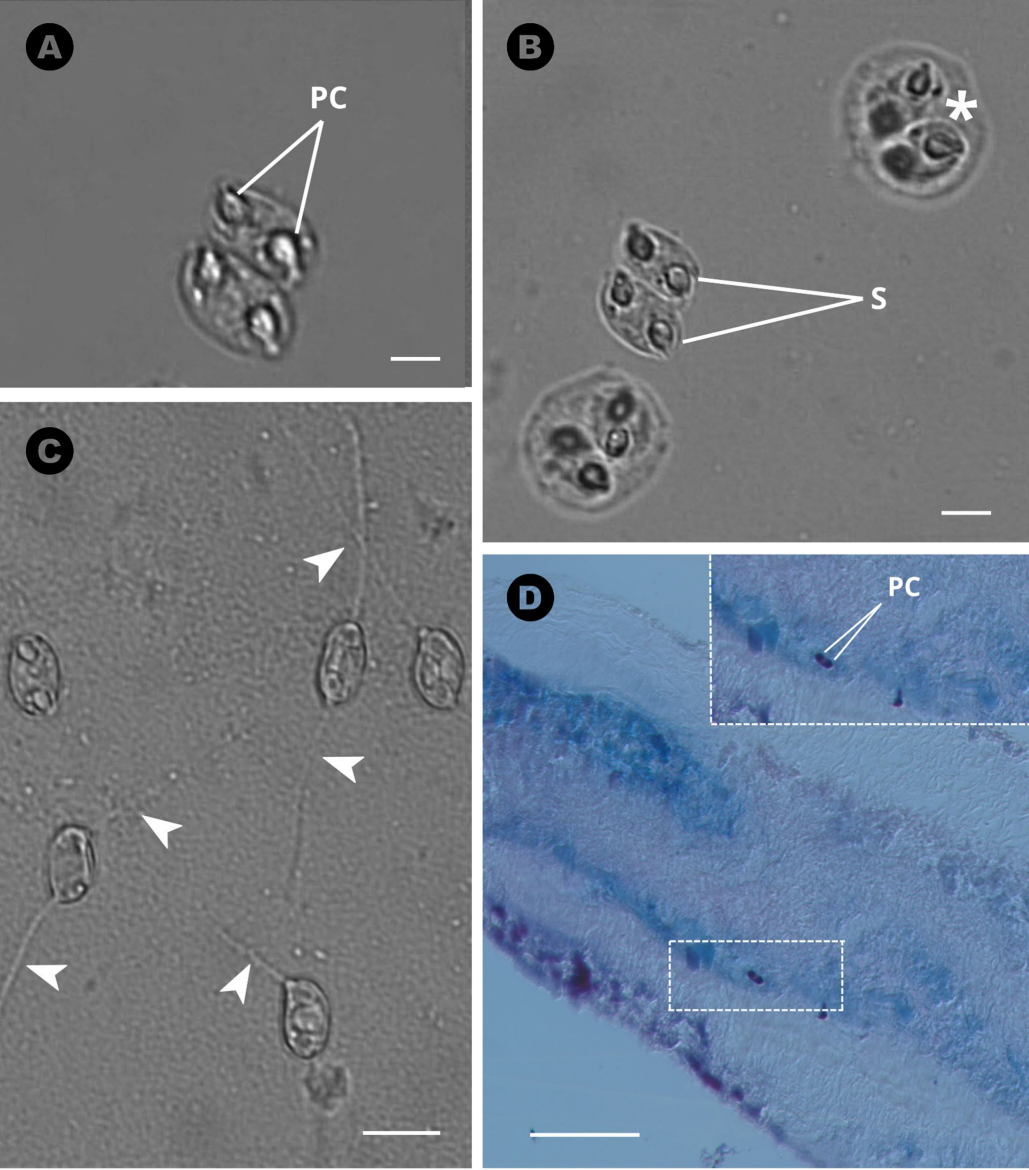
Light photomicrographs of *Ellipsomyxa colariensis* n. sp. in *Ageneiosus inermis*: (**A**) Mature spores, showing the polar capsules (**PC**) (scale bar = 5 μm); (**B**) Mature spores (S) of *E. colariensis* n. sp. found in the epithelium of the gallbladder and disporic plasmodia (*) (scale bar = 10 μm); (**C**) Spore undergoing extrusion of the polar filament (**arrowheads**) (scale bar = 5 μm); (**D**) Spore in the epithelium of the gallbladder (dashed rectangle), stained by the ZN technique (scale bar = 10 μm). Insert: detail of the spore showing its polar capsule

The mature *Ellipsomyxa colariensis* n. sp. spores were slightly elongated, with two spherical polar capsules of similar size located in the opposite sides of the spore (Fig. 2). Disporic plasmodia were also observed, with morphology consistent with the characteristics described for the genus *Ellipsomyxa* (Fig. 2b).

Mature spores are elongated, with an average length of 6.6 ± 0.5 (6.1–7.2) µm and a width of 4.0 ± 0.3 (3.6–4.1) µm. The two polar capsules (PC) found in the spore are similar in size and open subterminally in opposite directions, with an average length of 2.2 ± 0.1 (1.7–2.7) µm and a width of 1.9 ± 0.1 (2.1–1.8) µm (Table 1). It was not possible to determine the number of turns of the polar filaments.

**Table 1:** Comparison of the characteristics of the spores of the *Ellipsomyxa* species, including the new taxon described here (highlighted in bold), for which data are available

Species	Host	Country	Spore Morphology	Spore mean ± SD (µm) (Min – Max)		Polar Capsule mean ± SD (µm) (Min – Max)		References
				LS	WS	LP	WP	
***O Elipsomyxa colariensis*** **n. sp.**	***Ageneiosus inermis***	**Brasil**	**Elipsoid**	**6.6 ± 0.5** **(6.1–7.2)**	**4**,**0 ± 0.3** **(3**,**6–4**,**1)**	**2.2 ± 0.1** **(1.7–2.7)**	**1**,**9 ± 0.1** **(2.1–1.8)**	**Present study**
*Ellipsomyxa matosi*	*Ageneiosus ucayalensis*	Brazil	Elipsoid	13,1 ± 1,0 (11,9–14,4)	8,0 ± 0,8 (7,2–9,4)	4,9 ± 0,3 (4,4–5,4)	3.2 ± 0.6 (26–3.9)	[11]
*Ellipsomyxa tucujuensis*	*Satanoperca jurupari*	Brazil	Spherical	10,11 ± 0,86	7,81 ± 1,14	3,12 ± 0,53	2.5 ± 0.32	[19]
*Ellipsomyxa paraensis*	*Cichla monoculus*	Brazil	Elipsoid	11,5 (10,5– 12,4)	7,5 (6,6–8,6)	3.2 (2.1–3.9)	2.6 (2–3.3)	[20]
*Ellipsomyxa amazonensis*	*Brachyplatystoma rousseauxii*	Brazil	Elipsoid	12,80 (12,3– 13,6)	7,6 (6,7–8,7)	3,8 (3,8–4,0)	3.1 (2.5–3.4)	[21]
*Ellipsomyxa arariensis*	*Pigocentrus nattereri*	Brazil	Elipsoid	12,6 ± 0,5	7,3 ± 0,6	3,5 ± 0,2	2.6 ± 0.3	[22]
*Ellipsomyxa plagioscioni*	*Plagioscion* sp.	Brazil	Elipsoid	11.1 (10.2– 12.8)	6,6 (7,8 − 8,6)	3,8 (3,2–4,4)	2.8 (2.3–3.3)	[20]
*Ellipsomyxa gobioides*	*Gobioides broussonnetii*	Brazil	Elipsoid	6,8 (6,5–7,0)	7,2 (6,9–7,5)	4,6 (4,3–4,8)	2.5 (2.1–2.7)	[23]
*Ellipsomyxa santarenensis*	*Satanoperca jurupari*	Brazil	Elipsoid	12,0 (10,7–13,7)	7,6 (5,6–8,2)	2,8 (2,0–3,6)	2.8 (2.1–3.7)	[24]
*Ellipsomyxa ariusi*	*Ário ário*	India	Elipsoid	10.1 (9–12)	7,7 (7,1–8,7)	2,8 (2,1–3,7)	2.5 (1.6–3)	[25]
*Ellipsomyxa intravesica*	*Pangasius macronema*	Vietnam	Elipsoid	13,3 (12,0– 15,0)	8,4 (8,0–9,0)	3,7 (3,0–4,0)	3.6 (3.0–4.0)	[26]
*Ellipsomyxa kalthoumi*	*Liza Saliens*	Tunisia	Elipsoid	17.2 (13 − 21)	13.2 (10–15)	5,5 (5–6)	-	[27]
*Ellipsomyxa manilensis*	*Arothron manilensis*	Australia	Ovoid	15.2 (13.8–17.1)	11,8 (10,2–13,3)	5,6 (4,6–6,6)	4.5 (4.2–5.0)	[28]
*Ellipsomyxa arothroni*	*Arotron hispidus*	Australia	Ovoid	14,5 (11,3–16,0)	12.2 (9.4–13.8)	5,5 (4,5–6,7)	4.2 (3.1–5.0)	[28]
*Elipsomyxa* *Nigropunctatis*	*Arotron* *nigropunctatus*	Australia	Ovoid	13,8 (11,9–16,3)	9,9 (8,0–12,9)	4,7 (3,5–5,7)	3.6 (2.8–4.6)	[28]
*Ellipsomyxa apogoni*	*Apogon doederleini*	Australia	Elipsoid	10.2 (8.8–11.1)	6,9 (6,0–9)	3,7 (2,9–4,8)	2.7 (2.1–3.4)	[28]
*Ellipsomyxa adlardi*	*Gobiosoma bosc*	EUA	Elipsoid	12,4 (11,3–14,4)	7,7 (7,1–8,8)	4,3 (3,9–4,9)	3.6 (3.3–4.1)	[29]
*Ellipsomyxa syngnathi*	*Syngnathus* *rostellatus*	Denmark	Elipsoid	6,8 (6,3–7,2)	8.1 (7.2–8.6)	3.6 (3.2–4.1)	2.9 (2.7–3.2)	[30]
*Ellipsomyxa gobii*	*Pomatoschistus* *microps*	Denmark	Elipsoid	7,0 (6,6–7,5)	8,7 (8,0–9,0)	3.1 (3.0–3.2)	-	[9]
*Ellipsomyxa mugilis*	*Liza Saliens*	Spain	Ovoid	11,5 (10–13,5)	6,8 (5,5–8,0)	2,9 (2,7–4,0)	-	[31]
*Ellipsomyxa papantla*	*Dormitator maculatus*	Mexico	Ovoid	12,9 ± 0,8 (11,6–15,0)	9,1 ± 0,5 (7,6–9,9)	3,8 ± 0,5 (2,6–4,6)	3 ± 0.5 (2.2–4.2)	[32]
*Ellipsomyxa gordeyi*	*Gobiosoma bosc*	Vietnam	Elipsoid	9,5 ± 0,6 (8,7–10,9)	7,0 ± 0,5 (6,2–8,1)	3,5 ± 0,4 (2,7–4,3)	2.4 ± 0.2 (2.0–2.7)	[10]
*Ellipsomyxa prima*	*Gambusia yucatana*	Mexico	Elipsoid	9,5 ± 0,6 (8,3–10,9)	6,5 ± 0,5 (5,2–7,6)	3,2 ± 0,4 (2,5–4,5)	2.3 ± 0.2 (1.7–3.0)	[33]
*Ellipsomyxa filiformis*	*Hypophthalmus marginatus*	Brazil	Elipsoid	11.7 (10.2–13.4)	6.3 (7.4–5.2)	4.4 (3.8–4.8)	2.4 (2.2–2.8)	[34]
*Ellipsomyxa granulosa*	*Curimata inornata*	Brazil	Elipsoid	10.8 (10.2–11.7)	6.4 (5.7–7.1)	3.6 (3.2–4.2)	2.2 (2.0–2.5)	[34]

The parameters are: **LS** = Length of Spore; **WS** = Width of Spore; **LP** = Length of Polar capsule; **WP** = Width of Polar capsule

### Species - Taxonomic Summary

Phylum: Cnidaria Verrill, 1865;

Sub-phylum: Myxozoa Grassé 1970;

Class: Myxosporea Bütschli, 1881;

Order: Bivalvulida Shulman, 1959;

Family: Ceratomyxidae Doflein, 1899;

Genus: *Ellipsomyxa* Køie, 2003;

Species: *Ellipsomyxa colariensis* n. sp.

Type host: *Ageneiosus inermis* (Linnaeus, 1766);

Infection site: Epithelium of the gallbladder;

Type locality: Marajó Bay, off Humaitá Beach, located in the municipality of Colares, State of Pará, Brazil (0º56’30’’ S, 48º16’23’’ W).

Prevalence: 14 of the 25 (56%).

Histopathology: No histological alterations were observed in the analyzed fish.

Species deposition: A stained slide was deposited in the collection of Myxozoa of the National Amazonian Research Institute (INPA), in Manaus, Amazonas state, Brazil, under catalogue number INPA-CND 000109.

Molecular data: The partial sequence of the 18 S rDNA gene was deposited in GenBank under accession number PQ691409.

Etymology: The name of the species is derived from the collecting locality—the municipality of Colares, in the Brazilian state of Pará—of the host fish in which the parasite was identified.

### Phylogenetic and Molecular Analyses

A partial sequence of 1194 base pairs of the 18 S rDNA gene was obtained based on the sequencing of the DNA of the spores of *Ellipsomyxa colariensis* n. sp., found in the gallbladder of the host fish. This sequence was deposited in GenBank under accession number PQ691409.

**Fig. 3:**
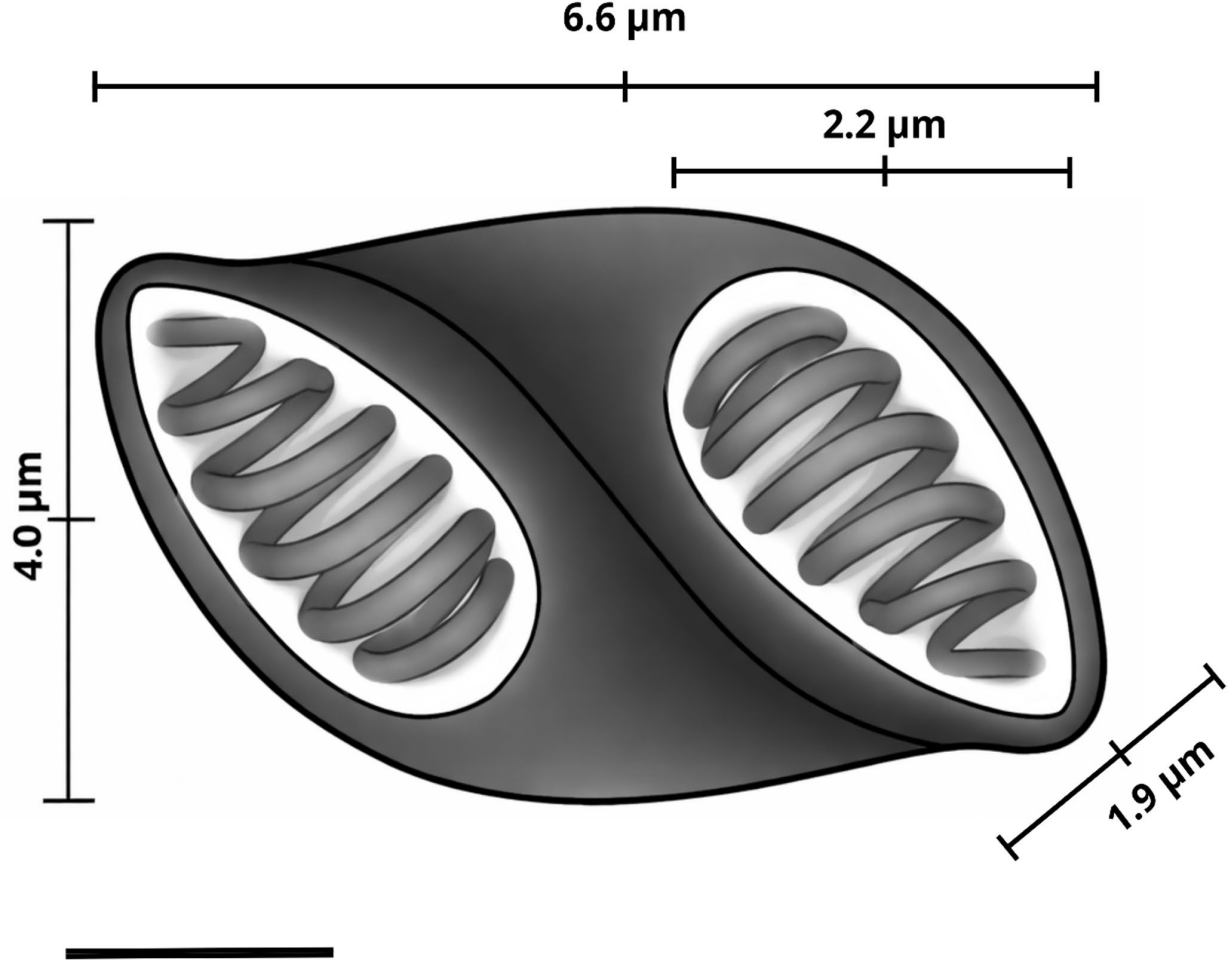
The Maximum Likelihood phylogenetic tree of the aligned partial sequences of the 18 S rDNA gene of *Ellipsomyxa colariensis* n. sp. and the other *Ellipsomyxa* and closely-related myxosporean species for which sequences are available. The GenBank accession number of each species follows its name. The numbers at the nodes are the reliability values. The new species described here is highlighted in bold type. The taxonomic order of the fish host is shown in the penultimate column on the left. **M =** Marine; **Est** = Estuarine; **F** = Freshwater

The Maximum Likelihood phylogenetic tree (Fig. 3) positions *Ellipsomyxa colariensis* n. sp. as an early-diverging species relative to the previously described Amazonian lineage of *Ellipsomyxa*, which includes *Ellipsomyxa amazonensis* Zatti [21], ; *Ellipsomyxa paraensis* [20]; *Ellipsomyxa santarenensis* [24]; *Ellipsomyxa arariensis* [22]; and *Ellipsomyxa matosi* [11].

The p-distance values (Table 2) estimated from 18 S rDNA sequences further support the species-level delimitation of *Ellipsomyxa colariensis* n. sp. The genetic divergence between *E. colariensis* n. sp. and the other analyzed species ranged from 0.040 to 0.060, which is consistent with interspecific differentiation within the genus *Ellipsomyxa*, considering the conservative nature of the 18 S rDNA marker in Myxozoa. The lowest divergence was observed in relation to *E. filiformis* (*p* = 0.040) and *E. amazonensis* (*p* = 0.044), Amazonian species that share the same infection site, suggesting close evolutionary relationships. Taken together, these results, combined with morphological, morphometric and phylogenetic evidence, support the recognition of *E. colariensis* n. sp. as a valid new species within the genus *Ellipsomyxa*.

**Table 2:** Genetic distances (*p*) recorded between *Ellipsomyxa colariensis* n. sp. and other *Ellipsomyxa* species from the Brazilian Amazon Basin

	Species (Acession Number in Genbank)	(1)	(2)	(3)	(4)	(5)	(6)		(7)
(1)	Ellipsomyxa colariensis *n*. sp (PQ691409)	–							
(2)	*Ellipsomyxa filiformis* (PX583100)	0.040	–						
(3)	*Ellipsomyxa granulosa* (PX583101)	0.048	0.020	–					
(4)	*Ellipsomyxa amazonensis* (MF193889)	0.044	0.032	0.031	–				
(5)	*Ellipsomyxa santarenensis* (OR142132)	0.045	0.045	0.033	0.001	-			
(6)	*Ellipsomyxa arariensis* (MH308206)	0.060	0.068	0.060	0.044	0.041	–		
(7)	*Ellipsomyxa paraensis* (MH364399)	0.045	0.033	0.030	0.003	0.001	0.041		–

## Discussion

*Ellipsomyxa colariensis* n. sp. clustered phylogenetically with *Ellipsomyxa filiformis* [34] and *Ellipsomyxa granulosa* [34]; however, it differs from these species in specific morphological characteristics of the plasmodia and spores. *E. filiformis* is distinguished by its elongated, filamentous plasmodia and by a less pronounced sutural curvature, in contrast to the ellipsoidal plasmodia observed in the present species. In turn, *E. granulosa* differs by the dense cytoplasmic granulation in its early developmental stages and by the characteristic angular inclination of the polar capsules relative to the spore axis. These morphological differences, together with the particularities of the host microenvironments, support the validity of *E. colariensis* n. sp. as a new taxon.

The presence of plasmodia containing two spores of *Ellipsomyxa colariensis* n. sp. in the gallbladder of the fish examined in the present study (Fig. 2B) is consistent with the previous observations of *E. arariensis* [22] and *Ellipsomyxa adlardi* [29] characteristics also found in these and in other previously described species. In *E. arariensis*, the plasmodia varied in size and shape, with ellipsoidal spores and a curved sutural line in sutural view. These similarities reinforce the morphological consistency within the genus *Ellipsomyxa*, including the species identified in the present study.

The extrusion of polar filaments was also observed in mature spores, a process also recorded in *Ellipsomyxa tucujuensis* [19] and in *E. gordeyi* [10]. These findings further highlight the morphological consistency among the species of the genus *Ellipsomyxa* and contribute to the taxonomic validation of the different forms, based on key structures, such as the extruded polar filaments.

Heiniger and Adlard (2014) highlighted that mature spores of the genus *Ellipsomyxa* may exhibit wide morphological variation, including ellipsoidal, pyriform, or ovoid shapes, depending on the species. In addition, Burger and Adlard (2010) reported that plasmodia may occur in either mono- or disporic forms. In the present study, the development of disporic plasmodia was observed (Fig. 2b), a pattern consistent with the records of Azevedo [23] for *E. gobioides* [5] and for *E. tucujuensis*. These plasmodia are characterized by a well-defined organization and a specific association with host tissues, reflecting a conserved pattern within the genus.

**Fig. 4:**
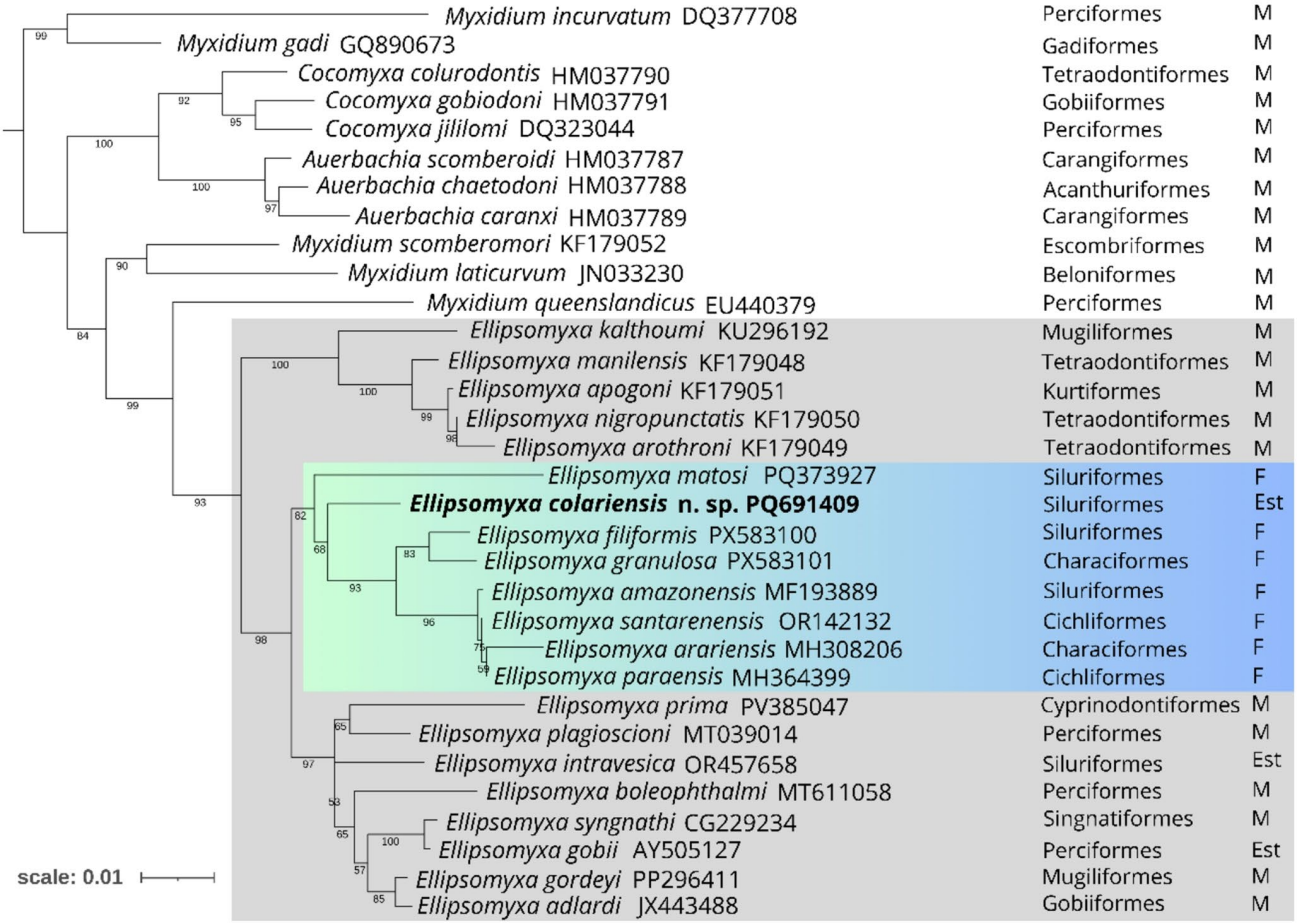
Diagram of the myxospore of *Ellipsomyxa colariensis* n. sp. found infecting *Ageneiosus inermis*, in the state of Pará, Brazil. Measurements show the length and width of the spore, as well as the length and width of the polar capsule. Image of the polar tubes is for illustrative purposes only. Scale bar = 5 μm

The spores observed in this study exhibited an ellipsoidal shape (Fig. 4), in accordance with the pattern described by Silva et al. (2021). Although Heiniger and Adlard (2014) reported that mature spores of the genus *Ellipsomyxa* can vary considerably in shape, including ellipsoidal, pyriform, or ovoid forms depending on the species, the morphology recorded herein falls within this known range of variation, reinforcing the morphological consistency of the species analyzed in relation to previously published descriptions. These findings support the identification of the parasite as a member of the genus *Ellipsomyxa* and indicate its occurrence in a new host, contributing to the understanding of the diversity and distribution of this group.

From a morphometric perspective, the spores of *Ellipsomyxa colariensis* n. sp. are most similar to those of *E. gobioides* in terms of length (Table 1). However, these species differ significantly in other characteristics, such as spore width and polar capsule length—the latter being notably larger in *E. gobioides*, reaching twice the length of those in the other species analyzed in this study. The absence of 18 S sequences for *E. gobioides* precludes a direct genetic comparison to support the divergence; nevertheless, phylogenetic analysis shows that *E. colariensis* n. sp. clusters with species that primarily infect freshwater hosts of the orders Siluriformes and Characiformes. Together, the morphometric and host differences reinforce its distinction from known species and support the description of this new species.

Although the lack of genetic data for *E. gobioides* limits a comparative phylogenetic analysis, the marked morphometric differences observed corroborate the identification of this new lineage. Regarding the pathological aspects, no apparent adverse effects of the infection were observed in the host fish, either in tissue coloration or the presence of visible external signs, despite the detection of mature spores in the gallbladder. This finding suggests that, under the evaluated conditions, the infection was of low intensity or did not cause evident macroscopic changes. The absence of manifestations contrasts with the findings of Azevedo [23], who described gallbladder hypertrophy in fish infected by *E. gobioides*, and Thabet [27], who documented epithelial necrosis in infections caused by *E. kalthomi*.

The lack of clear symptoms in the present study indicates that infection by *Ellipsomyxa colariensis* n. sp. does not result in discernible pathology under macroscopic analysis. We emphasize, however, that a detailed assessment of pathogenicity was not the primary focus of this study, which prioritized the taxonomic and phylogenetic description of the species. Therefore, further studies encompassing different parasite load profiles and host physiological responses are necessary to comprehensively characterize the pathogenic potential of this myxozoan and its impact on the health of Amazonian fish.

In this comparative context, *Ellipsomyxa matosi* also deserves attention, as it shares with *E. colariensis* n. sp. development in the gallbladder and the formation of disporic plasmodia containing mature spores. However, as described by Azevedo [23], *E. matosi* exhibits consistent differences in spore proportions and, particularly, in the dimensions of the polar capsules, which are relatively more elongated. In addition, subtle variations in the curvature of the sutural line contribute to the morphological distinction between these species. These differences highlight the morphological diversity within the genus, even among species that share the same site of infection.

Following the methodological recommendations of Yurakhno [10], the present study adopted a cautious approach to the inclusion of published DNA sequences of the species of the genus *Ellipsomyxa*. These authors highlighted possible inconsistencies in the sequences attributed to *Ellipsomyxa ariusi* [25], *E. arariensis* [22] and *E. tucujuensis* [19], observing problems in the alignment that were also perceived by Figueredo [24]. Given this, these sequences were examined carefully prior to their inclusion in the analyses presented here, and this screening confirmed these problems of alignment in *E. ariusi* and *E. tucujuensis*, which is why these two species were excluded from the phylogenetic analyses presented here. However, the sequence attributed to *E. arariensis* did not appear to have any inconsistencies, which permitted its inclusion in the phylogenetic analyses as a reliable representation of the species. This cautious approach was intended to guarantee robustness and reliability in evolutionary inferences based on the analysis of the DNA sequences.

*Ellipsomyxa colariensis* n. sp. was grouped phylogenetically with *E. matosi*,* E. filiformis*,* E. granulosa*, *E. amazonensis*, *E. santarenensis*, *E. arariensis* and *E. paraensis*, forming an exclusively Brazilian clade. All these species share the infection site in the gallbladder of their fish hosts, which indicates a conserved pattern of tissue tropism within the group. This grouping reinforces the evolutionary proximity among the Amazonian *Ellipsomyxa* species and indicates that the Amazon region as an important centre of diversity for the genus.

Considering the level of morphological, morphometric, and molecular differences observed in this study, *Ellipsomyxa colariensis* n. sp. is proposed as a new myxosporean species. This description of a new taxon represents the first record of the genus *Ellipsomyxa* infecting *A. inermis*. This discovery expands the known diversity of the genus *Ellipsomyxa*.

## Conclusions

The presence of a parasite of the genus *Ellipsomyxa* was confirmed in a catfish (*A. inermis*) collected along the Amazonian coast of Brazil. Comparisons of the morphological and morphometric characteristics of these parasites with those of other species of the genus *Ellipsomyxa*, together with the analysis of a partial fragment of the 18 S rDNA gene, allowed the assessment of the occurrence of a parasite belonging to the genus *Ellipsomyxa*.

The confirmation of A. inermis as a host species for a parasite of the genus *Ellipsomyxa* represents the first record of this genus infecting a fish of this species. This finding expands current knowledge of the diversity and distribution of microparasites in the Amazon region and highlights the need for further studies focused on the prospecting, identification, and characterization of parasites in this still poorly explored ecosystem.

## Data Availability

The DNA sequences are deposited in Genbank (PQ691409); a glass slide with spores stained with Ziehl-Neelsen (ZN) is deposited in the collection of the National Amazonas Research Institute (INPA), Manaus, Amazonas, Brazil (accession number: CND 000109). The data generated during the study are included in this article.
